# Severe Sepsis During Treatment for Childhood Leukemia and Sequelae Among Adult Survivors

**DOI:** 10.1001/jamanetworkopen.2024.2727

**Published:** 2024-03-18

**Authors:** Kathryn P. Goggin, Lu Lu, Danielle E. Lee, Carrie R. Howell, Deokumar Srivastava, Tara M. Brinkman, Gregory T. Armstrong, Nickhill Bhakta, Leslie L. Robison, Mathew J. Ehrhardt, Melissa M. Hudson, Kevin R. Krull, Ching-Hon Pui, Jeffrey Rubnitz, Kirsten K. Ness, Joshua Wolf

**Affiliations:** 1 Department of Infectious Diseases, St Jude Children’s Research Hospital, Memphis, Tennessee; 2 Now with Department of Pediatrics, Emory University School of Medicine and Children’s Healthcare of Atlanta, Atlanta, Georgia; 3 Department of Biostatistics, St Jude Children’s Research Hospital, Memphis, Tennessee; 4 Department of Pediatrics, University of Tennessee Health Science Center, Le Bonheur Children’s Hospital, Memphis; 5 Department of Psychology, St Jude Children’s Research Hospital, Memphis, Tennessee; 6 Department of Epidemiology and Cancer Control, St Jude Children’s Research Hospital, Memphis, Tennessee; 7 Department of Global Pediatric Medicine, St Jude Children’s Research Hospital, Memphis, Tennessee; 8 Department of Oncology, St Jude Children’s Research Hospital, Memphis, Tennessee

## Abstract

**Importance:**

Children undergoing treatment for leukemia are at increased risk of severe sepsis, a dysregulated immune response to infection leading to acute organ dysfunction. As cancer survivors, they face a high burden of long-term adverse effects. The association between sepsis during anticancer therapy and long-term organ dysfunction in adult survivors of childhood cancer has not been examined.

**Objective:**

To determine whether severe sepsis during therapy for leukemia in childhood is associated with subsequent chronic health conditions in adult survivors.

**Design, Setting, and Participants:**

This cohort study included 644 adult survivors of childhood leukemia who were diagnosed between January 1, 1985, and July 19, 2010, and participated in the St Jude Lifetime Cohort Study. Participants were excluded if they received hematopoietic cell transplant or had relapsed leukemia. Data collection ended June 30, 2017. Data were analyzed from July 1, 2020, to January 5, 2024.

**Exposures:**

Severe sepsis episodes, defined according to consensus criteria as septic shock, acute respiratory distress syndrome, or multiorgan dysfunction associated with infection occurring during anticancer therapy, were abstracted by medical record review for all participants.

**Main Outcomes and Measures:**

Common Terminology Criteria for Adverse Events–defined chronic health condition outcomes were independently abstracted. Associations between sepsis and cumulative incidence of chronic health conditions (eg, cardiovascular, pulmonary, kidney, neurological, and neurocognitive outcomes) were compared by adjusted hazard ratios from Cox proportional hazards logistic regression. Inverse propensity score weighting was used to adjust for potential confounders, including age, year of diagnosis, and leukemia type.

**Results:**

The study sample consisted of 644 adult survivors of pediatric leukemia (329 women [51.1%] and 315 men [48.9%]; including 56 with a history of acute myeloid leukemia and 585 with a history of acute lymphoblastic leukemia) who were most recently evaluated at a median age of 24.7 (IQR, 21.2-28.3) years at a median time after leukemia diagnosis of 17.3 (IQR, 13.7-21.9) years. Severe sepsis during treatment of acute childhood leukemia occurred in 46 participants (7.1%). Participants who experienced severe sepsis during treatment were more likely to develop moderate to severe neurocognitive impairment (29 of 46 [63.0%] vs 310 of 598 [51.8%]; adjusted hazard ratio, 1.86 [95% CI, 1.61-2.16]; *P* < .001) significantly affecting attention, executive function, memory and visuospatial domains. Sepsis was not associated with long-term risk of cardiovascular, pulmonary, kidney, or neurological chronic health conditions.

**Conclusions and Relevance:**

In this cohort study of long-term outcomes in survivors of pediatric leukemia, severe sepsis during anticancer therapy for leukemia was associated with a selectively increased risk for development of serious neurocognitive sequelae. Efforts to reduce the effects of anticancer therapy on long-term function and quality of life in survivors might include prevention of severe sepsis during therapy and early detection or amelioration of neurocognitive deficits in survivors of sepsis.

## Key Points

**Question:**

Is severe sepsis during treatment for childhood leukemia associated with subsequent development of chronic health problems in adult survivors?

**Findings:**

In 644 adult survivors of childhood leukemia, severe sepsis during treatment was associated with significantly higher rates of moderate to severe neurocognitive dysfunction. Sepsis was not associated with other identified chronic health conditions.

**Meaning:**

Severe sepsis during childhood cancer treatment might increase the risk of clinically meaningful long-term neurocognitive problems, but more research is needed to validate these findings, identify potential mechanisms, and develop ameliorative interventions.

## Teaser

This cohort study assesses whether severe sepsis during treatment for childhood acute leukemia is associated with increased incidence of long-term organ dysfunction among adult survivors using observational data from the St Jude Lifetime Cohort Study.

## Introduction

Approximately 2500 new cases of acute lymphoblastic leukemia (ALL) and 500 new cases of acute myeloid leukemia (AML) are diagnosed in children in the US annually.^1^ Long-term survival now exceeds 90% for children diagnosed with ALL^2,3^ and 70% for those with AML.^4^ A persistent threat to survival during leukemia therapy is the increased risk of infection, including bloodstream infection and sepsis, related to immunosuppression, myelosuppression, and the use of central venous catheters. Sepsis is life-threatening organ dysfunction due to a dysregulated host response to infection and has dramatic short-term effects on organ function.^5^ In 1 recent cohort, 29% of patients with ALL developed bacteremia, and 25% of patients with bacteremia developed sepsis.^6^ Nonfatal infections can result in permanent end-organ damage, contribute to chemotherapy delay or modification, and increase antibiotic exposure and the risk of selecting for drug-resistant bacteria.^7^ Evidence is emerging about the long-term end-organ and psychosocial effects of sepsis, but there are currently no specific guidelines for the follow-up or treatment of survivors of pediatric severe sepsis.^8^ In addition to the increased risk of sepsis and other acute treatment-related complications, survivors of pediatric cancer, including those treated for leukemia, have a higher burden of chronic health conditions as they age, both in number of morbidities and younger age at onset, compared with the general population.^9,10,11^

Many reports of survivors of pediatric leukemia have appropriately focused on the late effects of the cancer itself, chemotherapy, and other treatments needed to eradicate the cancer and the metabolic and endocrine complications that can arise during and after therapy.^10,11^ To date, the extent to which severe sepsis during therapy contributes to the long-term chronic health conditions associated with multiorgan dysfunction faced by leukemia survivors has not been explored. A 2018 study^6^ found that bacteremic sepsis during treatment for pediatric ALL was associated with impaired neurocognitive function in survivors about 5 years after completion of anticancer therapy.

We hypothesized that, given the known effects of severe sepsis on acute organ function, survivors who experienced severe sepsis during treatment for childhood acute leukemia would have increased incidence of long-term organ dysfunction compared with those who did not. We tested this hypothesis using observational data from the St Jude Lifetime Cohort Study (SJLIFE) and propensity score–adjusted models to reduce the effects of confounding factors in estimating causal effects. Defining the long-term implications of sepsis in this population could lead to increased implementation of interventions to prevent sepsis, including antibiotic prophylaxis, continuous ambulatory physiologic monitoring during induction therapy, and targeted use of novel diagnostics such as next-generation sequencing to predict and diagnose infection during anticancer treatment, in addition to increased monitoring for development of long-term organ dysfunction in cancer survivors who experienced this complication.

## Methods

### Study Design

This analysis used data from SJLIFE, a cohort study including patients treated at St Jude Children’s Research Hospital (SJCRH) with prospective follow-up and ongoing data accrual designed to assess health outcomes and reduce late effects among survivors of pediatric malignant neoplastic disease (eMethods in Supplement 1). Enrollment in the SJLIFE cohort began in 2007. The design and methodology for this institutional review board–approved study have been previously described; all study procedures and documents were approved by the SJCRH Institutional Review Board, and participants provided written informed consent prior to completion of any study measures.^12^ Medical record review was performed for all survivors of pediatric acute leukemia enrolled in SJLIFE to identify those who experienced severe sepsis during treatment and developed chronic health conditions prior to SJLIFE direct assessment. Medical record reviewers (K.P.G. and J.W.) were blind to organ status evaluations and neurocognitive outcome status. This study followed the Strengthening the Reporting of Observational Studies in Epidemiology (STROBE) reporting guideline.

### Participants

The population included all SJLIFE participants who had been diagnosed with leukemia between January 1, 1985, and July 19, 2010. Eligible survivors received all of their treatment for pediatric acute leukemia at SJCRH and/or affiliate locations, had a minimum survival of 5 years following completion of therapy, did not relapse or receive hematopoietic stem cell transplant, and completed an initial on-campus SJLIFE clinical assessment. Survivors who had completed an SJLIFE campus visit and subsequently died were eligible for the study. Patients at SJCRH who died prior to the inception of SJLIFE or living patients not enrolled in SJLIFE were ineligible for analysis. Medical events and vital status were abstracted from health records (medical reports, cancer registry follow-up, and next of kin contact, and/or the National Death Index for those lost to follow-up or deceased) by trained research staff.^13^ Outcomes included data available at the time of data freeze on June 30, 2017.

### Study Definitions

Episodes of severe sepsis were abstracted by direct medical record review, and additional case finding was performed by review of discharge coding and research databases. Sepsis was defined according to consensus criteria for severe sepsis and septic shock (eTables 1 and 2 in Supplement 1).^14^ Time to first event was defined as time to first Common Terminology Criteria for Adverse Events (CTCAE)–defined chronic health condition outcome.

### Outcomes

The primary outcomes established prior to data collection were cumulative incidence of specific end-organ chronic health conditions that could be plausibly associated with the occurrence of severe sepsis during leukemia treatment. Data on health conditions in the SJLIFE database were collected from medical records, health questionnaires, and in-person diagnostic assessments during SJLIFE on-campus follow-up visits and graded according to modified version 4.03 of the CTCAE; race and ethnicity data were self reported and were included because they are associated with leukemia outcomes and risk of infections.^15^ A list of conditions of interest and their respective grading criteria is provided in eTable 3 in Supplement 1. Secondary outcomes included development of neurocognitive impairment^6^ and development of sarcopenia (low muscle mass). Participants underwent a comprehensive neurocognitive assessment in standardized order that was administered by certified neurocognitive examiners under the supervision of a board-certified clinical neuropsychologist (eTable 4 in Supplement 1). Briefly, scores were referenced to national normative data to generate age-adjusted *z* scores for each neurocognitive outcome; impairment was defined by domain and categorized as CTCAE grade 1 for *z* scores −1.00 to −1.99, grade 2 for *z* scores −2.00 to −2.99, and grade 3 for *z* scores up to −3.00 as previously described.^15^ Neurocognitive examiners were not aware of the study hypothesis or sepsis status.

### Statistical Analysis

Data were analyzed from July 1, 2020, to January 5, 2024. The time scale used was the time of therapy end to the time of first CTCAE-defined event, censoring, or death. Cognitive impairment and sarcopenia were adjusted by time to first cognitive assessment or dual-energy x-ray absorptiometry scan to measure lean muscle mass. A weighted Cox proportional hazards regression model was used to investigate the association of sepsis with the subsequent risk of chronic health conditions. Adjusted hazard ratios (AHRs) were used to compare the cumulative risk of experiencing end-organ dysfunction, neurocognitive sequelae, or sarcopenia in patients who had experienced severe sepsis during treatment with those who had not. To account for possible confounding by variables that could affect the probability of both sepsis and the development of chronic health conditions, inverse propensity score weighting was used to adjust the model. Propensity scores were calculated using leukemia diagnosis (AML vs ALL), age at diagnosis, and year of diagnosis. An additional propensity score–weighted analysis comprising radiation exposure, age at and year of diagnosis, and cumulative doses of vincristine, intrathecal methotrexate, intravenous or oral methotrexate, high-dose methotrexate, alkylating agents, anthracyclines, asparaginase, corticosteroids, and epipodophyllotoxins was also performed. Because some participants had been included in a prior study evaluating the association between sepsis and neurocognitive dysfunction,^6,16^ a sensitivity analysis excluding these participants was also performed. Because we encountered several extreme weights, we calculated and used stabilized weights as recommended by Chesnaye et al.^17^ Model-based variance estimators were used.

All tests of statistical significance were 2 sided, with all null hypotheses tested at the 5% level of significance. To account for multiple testing, false discovery rate–controlling procedures were used. SAS, version 9.4 (SAS Institute Inc), was used for all statistical analyses. Two-sided *P* < .05 indicated statistical significance.

## Results

### Participant Characteristics

A total of 644 survivors of acute pediatric leukemia without relapse or progression to hematopoietic stem cell transplant who completed a SJLIFE campus evaluation were evaluable (329 women [51.1%] and 315 men [48.9%]). Participants were diagnosed with leukemia between January 1, 1985, and July 19, 2010. Median age at evaluation was 24.7 (IQR, 21.2-28.3) years, and median time from diagnosis was 17.3 (IQR, 13.7-21.9) years. In terms of race and ethnicity, 3 participants (0.5%) were Asian; 5 (0.8%), Hispanic; 88 (13.7%), non-Hispanic Black; 545 (84.6%), non-Hispanic White; and 3 (0.5%), unspecified. Of the 644 participants, 46 (7.1%) had at least 1 episode of severe sepsis during leukemia therapy. Septic shock was identified in 48 of 53 episodes of sepsis (90.6%), and acute sepsis-related encephalopathy was noted in 6 of 53 (11.3%). As shown in Table 1, important differences between groups were balanced after propensity score adjustment. A total of 16 of 56 participants (28.6%) with AML had experienced severe sepsis, compared with 30 of 585 (5.1%) with ALL (OR, 7.40 [95% CI, 3.73-14.70]).

**Table 1.** Demographic and Clinical Characteristics of Survivors of Childhood Leukemia^a^

Characteristic	Unadjusted			Propensity score adjusted		
	No sepsis group (n = 598)	Sepsis group (n = 46)	SMD	No sepsis group (n = 599.3)	Sepsis group (n = 43.0)	SMD
Sex						
Female	306 (51.2)	23 (50.0)	−0.024	306.9 (51.2)	21.5 (50.1)	−0.022
Male	292 (48.8)	23 (50.0)	0.024	292.4 (48.8)	21.5 (49.9)	0.022
Race and ethnicity						
Asian	3 (0.5)	0	−0.324	3.1 (0.5)	0	−0.100
Hispanic	5 (0.8)	0	−0.417	4.9 (0.8)	0	−0.127
Non-Hispanic Black	79 (13.2)	9 (19.6)	0.148	79.8 (13.3)	6.3 (14.7)	0.040
Non-Hispanic White	509 (85.1)	36 (78.3)	−0.177	508.6 (84.9)	36.4 (84.7)	−0.006
Unknown	2 (0.3)	1 (2.2)	−0.057	1.9 (0.3)	0.2 (0.6)	0.045
Leukemia type						
ALL	555 (92.8)	30 (65.2)	−0.720	543.1 (90.6)	39.0 (90.7)	0.003
AML	40 (6.7)	16 (34.8)	0.739	53.4 (8.9)	4.0 (9.3)	0.014
Other^b^	3 (0.5)	0	−0.324	2.8 (0.5)	0	−0.100
Craniospinal irradiation	150 (25.1)	5 (10.9)	−0.376	143 (24.0)	11 (25.6)	−0.048
Age at diagnosis, mean (SD), y	6.7 (4.8)	10.0 (5.6)	−0.627	6.97 (4.95)	5.68 (5.34)	0.251
Time to assessment, mean (SD), y^c^	17.8 (4.9)	15.1 (4.5)	0.565	17.57 (4.96)	17.2 (4.72)	0.076
Cumulative No. of chemotherapy doses, mean (SD)						
Vincristine	44.79 (23.00)	30.49 (25.18)	0.593	43.73 (23.52)	46.67 (20.40)	−0.134
Intrathecal methotrexate	197.63 (97.76)	120.06 (95.46)	0.803	192.02 (99.03)	223.13 (110.73)	−0.296
IV or oral methotrexate	1669.90 (1759.74)	1911.8 (2155.17)	−0.123	1685.77 (1786.64)	1691.48 (1808.91)	−0.003
High-dose methotrexate	13 025.85 (8881.89)	10 096.36 (8984.67)	0.328	12 806.23 (8943.15)	13 064.46 (8760.26)	−0.029
Alkylating agents^d^	4654.00 (4254.03)	2665.58 (3516.2)	0.510	4506.69 (4242.25)	5017.31 (4151.50)	−0.122
Anthracyclines^e^	113.53 (86.47)	200.83 (126.26)	−0.807	120.44 (95.84)	124.54 (86.87)	−0.045
Asparaginase^f^	8355.68 (8888.12)	8203.26 (10 092.60)	0.016	8324.25 (8970.24)	8505.25 (8128.84)	−0.021
Corticosteroids^g^	8908.96 (4896.77)	7163.45 (6181.63)	0.313	8769.63 (5025.83)	9473.56 (4691.15)	−0.145
Epipodophyllotoxins	44.79 (23.00)	30.49 (25.18)	0.593	6735.68 (6828.73)	7194.27 (6809.51)	−0.067

Abbreviations: ALL, acute lymphoblastic leukemia; AML, acute myeloid leukemia; IV, intravenous; SMD, standardized mean difference.

^a^ Unless otherwise indicated, data are expressed as No. (%) of patients.

^b^ Indicates mixed phenotype acute leukemia.

^c^ Indicates time since completion of cancer-directed therapy.

^d^ Given in cyclophosphamide equivalents.

^e^ Given in doxorubicin equivalents.

^f^ Given in pegylated equivalents.

^g^ Given in prednisone equivalents.

### Outcomes

There was no significant difference in the cumulative incidence of clinically assessed chronic cardiovascular, pulmonary, kidney, or neurological conditions between survivors with or without severe sepsis during therapy (Table 2). There was also no significant difference in prevalence of sarcopenia between these 2 groups. An analysis that included other potentially confounding variables in addition to those defined in the a priori analysis plan confirmed these results (eTable 5 in Supplement 1).

**Table 2.** Incidence of Chronic Health Conditions by History of Severe Sepsis During Therapy for Childhood Leukemia^a^

Chronic condition	No. (%) of patients		*P* value
	No sepsis (n = 598)	Sepsis (n = 46)	
Cardiopulmonary			
Cardiomyopathy, right ventricular systolic dysfunction, right heart failure, myocardial infarction	14 (2.3)	0	.55
Restrictive pulmonary deficit, pulmonary diffusion deficit	4 (0.7)	0	.60
Severe restrictive or obstructive pulmonary deficit, pulmonary diffusion deficit (grade 3 or higher)	3 (0.5)	0	.78
Kidney			
Severe chronic kidney disease (grade 3 or higher)	2 (0.3)	0	.81
Neurological			
Cerebrovascular accident	6 (1.0)	0	.73
Cerebrovascular accident, neuromuscular disorder	6 (1.0)	0	.73
Severe cerebrovascular accident, neuromuscular disorder, seizures (grade 3 or higher)	14 (2.3)	0	.54
All primary analysis events (cardiac, pulmonary, neurological) combined	22 (3.7)	0	.43
Sarcopenia (low muscle mass)	170 (28.4)	14 (30.4)	.89

^a^ Models are adjusted for propensity score weights including diagnosis (acute myeloid leukemia vs acute lymphoblastic leukemia), age at diagnosis, and year of diagnosis.

By contrast, survivors who experienced severe sepsis during treatment were significantly more likely to have any moderate to severe neurocognitive impairment (29 of 46 [63.0%] vs 310 of 598 [51.8%]; AHR, 1.86 [95% CI, 1.61-2.16]; *P* < .001) (Table 3). Specifically, they were at higher risk of moderate to severe attention (19 of 46 [41.3%] vs 143 of 598 [23.9%]; AHR, 2.37 [95% CI, 1.93-2.91]; *P* < .001), memory (19 of 46 [41.3%] vs 158 of 598 [26.4%]; AHR, 2.72 [95% CI, 2.23-3.34]; *P* < .001), executive function (22 of 46 [47.8%] vs 187 of 598 [31.3%]; AHR, 2.28 [95% CI, 1.91-2.73]; *P* < .001), and visuospatial (8 of 46 [17.4%] vs 68 of 598 [11.4%]; AHR, 1.86 [95% CI, 1.39-2.48]; *P* < .001) impairment. They were also more likely to have some lower grades of impairment. An analysis including other potentially confounding variables in addition to those defined in the a priori analysis plan confirmed these results (eTable 6 in Supplement 1). Participants who had acute encephalopathy during sepsis did not appear more likely to have moderate to severe neurocognitive impairment (2 of 6 participants [33.3%] with sepsis-associated encephalopathy had any moderate to severe cognitive impairment compared with 27 of 40 [67.5%] without sepsis-associated encephalopathy).

**Table 3.** Neurocognitive Impairment by History of Severe Sepsis During Therapy for Childhood Leukemia

Neurocognitive condition	No. (%) of patients		Unadjusted model		Adjusted model^a^	
	No sepsis (n = 598)	Sepsis (n = 46)	HR (95% CI)	*P* value	HR (95% CI)	*P* value
**Moderate to severe impairment (grades 2-3)**						
Attention impairment	143 (23.9)	19 (41.3)	2.40 (1.47-3.91)	.001	2.37 (1.93-2.91)	<.001
Processing speed deficit	41 (6.9)	3 (6.5)	1.33 (0.40-4.37)	.64	0.91 (0.61-1.45)	.78
Memory impairment	158 (26.4)	19 (41.3)	2.22 (1.35-3.64)	.002	2.72 (2.23-3.34)	<.001
Executive function impairment	187 (31.3)	22 (47.8)	1.79 (1.08-2.99)	.02	2.28 (1.91-2.73)	<.001
Visuospatial impairment	68 (11.4)	8 (17.4)	1.77 (1.20-2.61)	.004	1.86 (1.39-2.48)	<.001
Any moderate to severe cognitive impairment	310 (51.8)	29 (63.0)	1.77 (1.19-2.61)	.004	1.86 (1.61-2.16)	<.001
**Any impairment (grades 1-3)**						
Attention impairment	257 (43.0)	22 (47.8)	1.80 (1.16-2.78)	.009	1.44 (1.22-1.70)	<.001
Processing speed deficit	147 (24.6)	16 (34.8)	1.60 (0.85-3.00)	.14	1.80 (1.47-2.21)	<.001
Memory impairment	333 (55.7)	29 (63.0)	1.78 (1.14-2.78)	.01	1.71 (1.48-1.97)	<.001
Executive function impairment	370 (61.9)	30 (65.2)	1.96 (0.09-4.25)	.09	1.59 (1.39-1.83)	<.001
Visuospatial impairment	108 (18.1)	8 (17.4)	1.56 (0.82-2.95)	.18	1.31 (1.01-1.69)	.04
Any cognitive impairment	499 (83.4)	39 (84.8)	1.57 (1.08-2.30)	.02	1.37 (1.22-1.56)	.02

Abbreviation: HR, hazard ratio.

^a^ Adjusted for propensity score weights including diagnosis (acute myeloid leukemia vs acute lymphoblastic leukemia), age at diagnosis, and year of diagnosis.

## Discussion

To our knowledge, this is the first study to address the potential for long-term associations between severe sepsis during therapy for pediatric leukemia and chronic health conditions in multiple body systems. Since severe sepsis is associated with acute multiorgan injury, we hypothesized that this damage could predispose patients to long-term complications in those systems. Consistent with previous data, SJLIFE participants who had experienced severe sepsis had a significantly increased risk of long-term neurocognitive dysfunction that could limit function and quality of life. Contrary to our original hypothesis, chronic conditions in other organ systems did not appear to be associated with sepsis, with no increase in risk of chronic cardiac, pulmonary, kidney, or neurological problems observed in those with severe sepsis during therapy.

This study adds to previously reported findings of poorer long-term neurocognitive outcomes in pediatric patients who experience severe sepsis during leukemia therapy using outcomes from a larger cohort with longer follow-up from cancer therapy.^6,16^ The cohort of pediatric cancer survivors followed up at SJCRH constitutes a unique dataset. Survivors of treatment for childhood cancer are already known to have poorer neurocognitive performance than their unaffected peers,^18^ and a recent study showed that experiencing bacteremic sepsis during leukemia treatment was associated with declining attention performance over time.^16^ Here, we show that the increase in attention impairment and higher-grade neurocognitive dysfunction do not appear driven by higher proportions of neurological conditions such as cerebrovascular accidents or seizures among those with sepsis. This finding is important because neurocognitive function is a priority for patients, caregivers, and clinicians, and neurocognitive dysfunction in survivors of childhood cancer is associated with significant reductions in rates of college graduation, employment, and income.^19^ A recent study of parental and physician treatment priorities found that avoidance of severe neurocognitive impairment was the predominant driver of treatment preferences when the risks of late effects were considered.^20^

The mechanism for the association is not known; sepsis-related encephalopathy can lead to permanent neurocognitive dysfunction and functional impairments and may increase the risk of vascular and psychiatric disease, but this exposure was rare in our cohort and did not seem to explain the identified neurocognitive defects.^21^ An alternative explanation is that cerebral damage might be potentiated in children with severe sepsis by increased exposure to neurotoxic chemotherapy drugs during periods of sepsis-induced increases in blood-brain barrier permeability.^6^ Our CTCAE grading for neurocognition in this report is consistent with the previously published grading approach using a modification of the CTCAE, version 4.03.^15^ These grades are based on population normative data so that a *z* score of up to −3.00 represents the lowest 0.13% of the population; a *z* score of up to −2.00 and greater than −3.00 represents the next highest 2.15% of the population; and a *z* score of up to −1.00 and greater than −2.00 represents the next highest 13.59% of the population. Persons in these categories are thus in the lowest 15.87% of the population. A *z* score of up to −1.00 also corresponds to an intelligence quotient of 85 or less where the population mean for is 100.

The lack of any identified association between severe sepsis and long-term organ dysfunction might be related to survivorship bias, missing complications, or complete recovery from sepsis-related injury. Because participation in the SJLIFE cohort requires individuals to be living 5 years or longer after diagnosis, it is possible that survivorship bias, or those with greatest organ dysfunction not surviving a septic event, could explain the lack of association with long-term chronic health conditions observed in our study.^22^ Although complications could have been missing from the dataset, the cumulative incidences of serious events are consistent with previously published data from larger SJCRH cohorts.^23^ Additionally, follow-up may have been insufficient for the development of these chronic health conditions.

### Limitations

This study has some limitations. Although patients included in the study were treated in different decades on evolving protocols, children treated for leukemia in the current era receive many of the same therapies and still face risk of sepsis, and important exposure variables were controlled by inverse propensity score–weighted analysis. There are numerous potential alternative causes for chronic outcomes after cancer, and baseline neurocognitive function data were not available. Patients who experienced severe cardiopulmonary long-term effects related to sepsis might have had increased risk of relapse or decreased survival and may be underrepresented in the study cohort. Only 6 patients in the study experienced sepsis-associated encephalopathy, so this is unlikely to be the cause of the neurocognitive differences. Some participants were included in a previous study of neurocognitive function after sepsis^6^; however, excluding them did not affect the association. As SJLIFE follow-up visits occur only every 3 to 5 years, events may have occurred since the last visit. For some outcomes used in the analysis, such as frailty or low muscle mass, the date of a graded event is likely to coincide with the date of the SJLIFE assessment. For undiagnosed and subclinical health conditions identified during research assessments, this may be more likely to introduce recall bias. However, the method of grading within each condition is consistent, and similarly graded conditions were grouped for analysis.^24^

## Conclusions

In this cohort study of long-term outcomes in adult survivors of pediatric leukemia, severe sepsis during anticancer therapy for leukemia was selectively associated with long-term, clinically relevant neurocognitive dysfunction, but not other persistent organ dysfunction. Prevention of severe sepsis during therapy, as well as recognition and amelioration of neurocognitive deficits through early intervention, might improve quality of life in children who survive childhood cancer.

## Notes

Supplement 1. **eMethods.** Data Collection **eTable 1.** Definition of Severe Sepsis (Includes Septic Shock) **eTable 2.** Organ Dysfunction Criteria for Classification of Severe Sepsis **eTable 3.** Chronic Health Conditions and CTCAE Grade Definitions **eTable 4.** Neuropsychological Health Event Grading in the St Jude Lifetime Cohort Study **eTable 5.** Incidence of Chronic Health Conditions by History of Severe Sepsis During Therapy for Leukemia: Alternative Analysis Approach **eTable 6.** Neurocognitive Impairment by History of Severe Sepsis During Therapy for Leukemia: Alternative Analysis Approach
eReferences

Supplement 2.
Data Sharing Statement
