# Supplementary material for: Severe Sepsis During Treatment for Childhood Leukemia and Sequelae Among Adult Survivors
Source: JAMA Netw Open. 2024 Mar 18;7(3):e242727. doi: 10.1001/jamanetworkopen.2024.2727 (PMC10949094; doi:10.1001/jamanetworkopen.2024.2727)
Supplement: Supplement 1. — eMethods. Data Collection eTable 1. Definition of Severe Sepsis (Includes Septic Shock) eTable 2. Organ Dysfunction Criteria for Classification of Severe Sepsis eTable 3. Chronic Health Conditions and CTCAE Grade Definitions eTable 4. Neuropsychological Health Event Grading in the St Jude Lifetime Cohort Study eTable 5. Incidence of Chronic Health Conditions by History of Severe Sepsis During Therapy for Leukemia: Alternative Analysis Approach eTable 6. Neurocognitive Impairment by History of Severe Sepsis During Therapy for Leukemia: Alternative Analysis Approach eReferences [file jamanetwopen-e242727-s001.pdf]

Supplementary Online Content

Goggin KP, Lu L, Lee DE, et al. Severe sepsis during treatment for childhood leukemia and sequelae among adult survivors. *JAMA Netw Open*. 2024;7(3):e242727. doi:10.1001/jamanetworkopen.2024.2727

eMethods. Data Collection

eTable 1. Definition of Severe Sepsis (Includes Septic Shock)

eTable 2. Organ Dysfunction Criteria for Classification of Severe Sepsis

eTable 3. Chronic Health Conditions and CTCAE Grade Definitions

eTable 4. Neuropsychological Health Event Grading in the St Jude Lifetime Cohort Study

eTable 5. Incidence of Chronic Health Conditions by History of Severe Sepsis During Therapy for Leukemia: Alternative Analysis Approach

eTable 6. Neurocognitive Impairment by History of Severe Sepsis During Therapy for Leukemia: Alternative Analysis Approach

eReferences

This supplementary material has been provided by the authors to give readers additional information about their work.

© 2024 Goggin KP et al. *JAMA Network Open*.

eMethods. Data Collection

Data on health conditions in the SJLIFE database are collected from medical records, health questionnaires, and in-person diagnostic assessments during SJLIFE on-campus follow-up visits and graded according to a modified Common Terminology Criteria for Adverse Events, as previously described.[1-4]

Neurocognitive dysfunction was also graded according to the modified CTCAE, with Z-scores based on population normative data, so that a Z-score of ≤-3 (Grade 3) represents the lowest 0.13% of the population; a Z-score ≤ -2 and > -3 (Grade 2) the next highest 2.15%; and a Z-score of ≤ -1 and > -2 (Grade 1) the next highest 13.59%, as previously described.[3]

Survivors who had completed a SJLIFE campus visit but subsequently died were eligible, but those who died prior to inception of SJLIFE or living patients not enrolled in SJLIFE were ineligible.

Sepsis episodes occurring during therapy for leukemia were classified using modified consensus criteria as shown in eTables 1 and 2.[5] Because blood gas measurements were not routinely available, and availability bias might affect the classification of sepsis, either PaO_2 or SaO_2 were used for evaluation.[6] Chart reviewers were not aware of long-term chronic conditions or of neurocognitive outcomes at the time of data collection or sepsis classification.

© 2024 Goggin KP et al. *JAMA Network Open*.

eTable 1. Definition of Severe Sepsis (Includes Septic Shock)[5]

**Severe sepsis:**<br><br>Sepsis (systemic inflammatory response syndrome plus suspected or proven infection) plus one of the following: cardiovascular organ dysfunction OR acute respiratory distress syndrome OR 2 or more other organ dysfunctions
**Septic shock:**<br><br>Sepsis plus cardiovascular organ dysfunction

Adapted from Goldstein B, Giroir B, Randolph A, et. al, “International pediatric sepsis consensus conference: definitions for sepsis and organ dysfunction in pediatrics”

© 2024 Goggin KP et al. *JAMA Network Open*.

eTable 2. Organ Dysfunction Criteria for Classification of Severe Sepsis[5,6]

Cardiovascular dysfunction:

Despite administration of isotonic intravenous fluid bolus ≥40 mL/kg in 1 hour:

- Decrease in blood pressure (BP) <5th percentile for age or systolic BP <2 SD below normal for age
  OR
- Need for vasoactive drug to maintain BP in normal range (dopamine >5 µg/kg/min or dobutamine, epinephrine, or norepinephrine at any dose)
  OR
- *Two of the following:*
  Unexplained metabolic acidosis: base deficit >5.0 mEq/L
  Increased arterial lactate >2 times upper limit of normal (ULN)
  Oliguria: urine output <0.5 mL/kg/hr
  Prolonged capillary refill: >5 seconds

Respiratory dysfunction:

- PaO_2/FiO_2 <300 in absence of cyanotic heart disease or preexisting lung disease
  OR
- $PaCO_2$ >65 torr or 20 mm Hg over baseline $PaCO_2$
  OR
- Proven need or >50% FiO_2 to maintain saturation ≥ 92%
  OR
- Need for nonelective invasive or noninvasive mechanical ventilation

ARDS: must include PaO_2/FiO_2 ratio ≤ 200 mm Hg, bilateral infiltrates, acute onset, and no evidence of left heart failure. If arterial blood gas was not obtained, SpO2/FiO2 ≤213 (if SpO2 <97%) could be substituted.[6]

Neurologic dysfunction:

- Glasgow Coma Score (GCS) ≤ 11
  OR
- Acute change in mental status with a decrease in GCS ≥ points from an abnormal baseline
  OR
- Altered mental status, not otherwise explained*

Hematologic dysfunction*:

- International normalized ratio >2
  OR
- Disseminated intravascular coagulation (clinical findings and abnormal coagulation studies)

Renal dysfunction:

- Serum creatinine ≥ 2 times ULN for age or 2-fold increase in baseline creatinine beyond ULN
  OR
- Required dialysis

Hepatic dysfunction:

- Total bilirubin ≥ 4 mg/dL
  OR
- Alanine transaminase (ALT, SGPT) 2 x ULN for age (and doubling if pre-sepsis value abnormal)

*Altered mental status was added due to frequency of GCS not being recorded. **Thrombocytopenia was excluded from criteria due to confounding secondary to leukemia therapy.

© 2024 Goggin KP et al. *JAMA Network Open*.

eTable 3. Chronic Health Conditions and CTCAE Grade Definitions[3]

CARDIOVASCULAR ORGAN SYSTEM:

Cardiomyopathy (encompasses decreased ejection fraction and LV systolic dysfunction)
Definition cardiomyopathy (event): EF < 50%, SF < 28% by echocardiography and/or MUGA based
Grades:
1: Not applicable
2: Resting EF <50-40%; 10 - 19% absolute drop from baseline
3: Resting EF 39-20%; >20% absolute drop from baseline; medication initiated or initiated
4: Resting EF<20%; refractory or poorly controlled heart failure due to drop in ejection fraction; intervention such as ventricular assist device, intravenous vasopressor support, or heart transplant indicated
5: Death

Right ventricular systolic (encompasses right ventricular systolic dysfunction; grades 2-5 are same for cor pulmonale)
Definition (event): right ventricular systolic dysfunction
Grades:
1: Asymptomatic cardiac imaging abnormalities
2: Symptoms with mild to moderate activity or exertion
3: Severe symptoms, associated with hypoxia, right heart failure; oxygen indicated
4: Life-threatening consequences; urgent intervention indicated (e.g., ventricular assist device); heart transplant indicated
5: Death

Right heart failure
Definition (event): cor pulmonale (right heart failure)
Grades (2-5 same as RV systolic dysfunction):
1: Asymptomatic with laboratory(e.g., BNP) or cardiac imaging abnormalities
2: Symptoms with mild to moderate activity or exertion
3: Severe symptoms, associated with hypoxia, right heart failure; oxygen indicated
4: Life-threatening consequences; urgent intervention indicated (e.g., ventricular assist device); heart transplant indicated
5: Death

Pulmonary hypertension
Definition (event): Pulmonary hypertension (confirmed by cardiac catheterization)
Grades:
1: Asymptomatic or mild symptoms; clinical or diagnostic observations only; intervention not indicated
2: Moderate; minimal, local or noninvasive intervention indicated; limiting age appropriate instrumental ADL
3: Severe or medically significant but not immediately life threatening; hospitalization or prolongation of existing hospitalization indicated; disabling; limiting self-care ADL
4: Life-threatening consequences; urgent intervention indicated
5: Death

Myocardial infarction
Definition (2 events for same grading rubric): Acute myocardial infarction, Coronary artery disease
Grades:
1: Not applicable
2: Asymptomatic and cardiac enzymes minimally abnormal and no evidence of ischemic ECG changes
3: Severe symptoms; cardiac enzymes abnormal; hemodynamically stable; ECG changes consistent with infarction (Q waves)

© 2024 Goggin KP et al. *JAMA Network Open*.

4: Life-threatening consequences; hemodynamically unstable (CABG or angioplasty)
5: Death

Hypertension
Definition (event): Based on BP measurements from the Human Performance lab
Grades:
1: Prehypertension (systolic BP 120 - 139 mm Hg or diastolic BP 80 - 89 mm Hg) from resting BP in HPL.
2: Stage 1 hypertension (systolic BP 140 - 159 mm Hg or diastolic BP 90 - 99 mm Hg); medical intervention indicated **or initiated**; recurrent or persistent (>=24 hrs); symptomatic increase by >20 mm Hg (diastolic) or to >140/90 mm Hg if previously WNL; monotherapy indicated or initiated
Pediatric: recurrent or persistent (>=24 hrs) BP >ULN; monotherapy indicated or initiated
3: Stage 2 hypertension (systolic BP >=160 mm Hg or diastolic BP >=100 mm Hg); medical intervention indicated; more than one drug or more intensive therapy than previously used indicated or initiated
Pediatric: Same as adult
4: Life-threatening consequences (e.g., malignant hypertension, transient or permanent neurologic deficit, hypertensive crisis); urgent intervention indicated
5: Death
Pediatric: Same as adult

PULMONARY ORGAN SYSTEM:

Obstructive pulmonary deficit
Definition (event): Chronic obstructive pulmonary disease
Includes: emphysema, chronic bronchitis, and chronic obstructive asthma
Grades:
1: Asymptomatic or mild symptoms; clinical or diagnostic observations only; intervention not indicated
2: Moderate; minimal, local or noninvasive intervention indicated or initiated (inhaled medications); limiting age- appropriate instrumental ADL
3: Severe or medically significant but not immediately life-threatening (e.g., requiring supplementation of oxygen, systemic corticosteroids, BIPAP, or CPAP); hospitalization or prolongation of existing hospitalization indicated; disabling; limiting self-care ADL
4: Life-threatening consequences; urgent intervention indicated
5: Death

Restrictive pulmonary deficits
Definition (event): Total lung capacity (TLC) decreased. Defined by TLC < 75**%.**
Grades:
1: TLC: 74 -70%
2: TLC: 69 - 60% predicted; limiting instrumental ADL
3: TLC: <60% predicted; limiting self-care ADL
4: Not applicable
5: Not applicable

Pulmonary diffusion deficits
Definition (event): Pulmonary diffusion decreased. Defined by DLCO < 75%.
Grades:
1: DLCO: 74-60% predicted
2: DLCO: <60%-40% predicted
3: DLCO: <40% predicted
4: Not applicable
5: Not applicable

RENAL SYSTEM:

© 2024 Goggin KP et al. *JAMA Network Open*.

Chronic kidney disease
Definition (event): Chronic kidney disease. Includes CKD requiring dialysis/transplant.
Grades:
1: eGFR (estimated Glomerular Filtration Rate) or CrCl (creatinine clearance) <LLN – 60 ml/min/1.73 m2 AND proteinuria 1+ present; urine protein/creatinine >0.5
2: eGFR or CrCl 59-30 ml/min/1.73 m2; salt-wasting requiring electrolyte replacement
3: eGFR or CrCl 29-15 ml/min/1.73 m2
4: eGFR or CrCl <15 ml/min/1.73 m2; dialysis or renal transplant indicated/performed
5: Death

NEUROLOGIC SYSTEM:

Cerebrovascular accident
Definition (event): Cerebrovascular accident (Includes: lacunes, hemorrhagic stroke, Ischemic stroke)
Code acute event grade based on CTCAE
Assess MRS at clinic eval
Grades:
1: Asymptomatic or mild neurologic deficit; radiographic findings only
2: Moderate neurologic deficit
3: Severe neurologic deficit
4: Life-threatening consequences; urgent intervention indicated
5: Death

Neuromuscular disorder
Definition (event): Neuromuscular disorders
Includes: dermatomyositis, myotonia, neuromuscular disorder, polymyositis, rhabdomyolysis, steroid myopathy, **critical illness myopathy/neuropathy**
Grades:
1: Asymptomatic or mild symptoms; clinical or diagnostic observations only; intervention not indicated
2: Moderate; minimal, local or noninvasive intervention indicated; limiting age appropriate instrumental ADL
3: Severe weakness or sensory loss; limiting self-care ADL
4: Life-threatening consequences; urgent intervention indicated
5: Death

Seizures
Definition (event): seizures
Grades:
1: Seizures not requiring medication
2: Seizures requiring one non-prn medication
3: Seizures requiring 2 or more non-prn medications; poorly controlled seizures with prescribed medications
4: Seizures requiring surgical intervention
5: Death

SARCOPENIA:

Sarcopenia is graded as present or absent and is defined as having a relative lean mass Z-score lower than 1 standard deviation of the mean from the same age, gender and race group in normal population (NHANES).

© 2024 Goggin KP et al. *JAMA Network Open*.

eTable 4. Neuropsychological Health Event Grading in the St Jude Lifetime Cohort Study[3]

Domain	Grading Source	Grading Rubric
Attention deficit	Performance on neuropsychological testing of attention, including sustained attention and focused attention. Impairment not explained by slow processing speed.	1: Performance on a task is > 1 but < 2 SD below the mean AND no functional impairment<br>2: Participant is prescribed medication to address attention impairment (i.e. stimulant); performance on a task is > 2 but < 3 SD below the mean OR performance on a task is > 1 but < 2 SD below the mean AND functional impact on instrumental activities. Examples include, but are not limited to: special education services at school (IEP, 504 plan, NOT Self-contained), unable to adequately complete testing due to attention impairments, difficulty in educational/occupational settings due to attention<br>3: Performance on a task is > 3 SD below the mean OR performance on a task is > 1 but < 3 SD below the mean AND functional impact on self-care activities. Examples include, but are not limited to: unable to live independently due to inattention, unable to work due to inattention, self-contained classroom<br>4: Not applicable<br>5: Not applicable<br><br>*Note: we do not code current medication for attention, but do indicate next to the assigned grade.
Processing speed deficit	Performance on neuropsychological testing of information processing speed and fluency. Fluency problems must not be accompanied by impairment on other measures of executive function.	1: Performance on a task is > 1 but < 2 SD below the mean AND no functional impairment<br>2: Performance on a task is > 2 but < 3 SD below the mean OR performance on a task is > 1 but < 2 SD below the mean AND functional impact on instrumental activities. Examples include, but are not limited to: special education services at school (IEP, 504 plan, NOT Self-contained), unable to reach educational/occupational goals secondary to cognitive impairment, Assistance needed completing tasks at home, scheduling/ attending appointments<br>3: Performance on a task is > 3 SD below the mean OR performance on a task is > 1 but < 3 SD below the mean AND functional impact in self-care activities. Examples include, but are not limited to: unable to live independently, unable to work, self-contained classroom<br>4: Not applicable<br>5: Not applicable
Memory deficit	Performance on neuropsychological testing of memory, including short-term verbal memory, new verbal learning, delayed verbal memory, and visual memory. Impairment not explained by attention deficits.	1: Performance on a task is > 1 but < 2 SD below the mean AND no functional impairment<br>2: Performance on a task is > 2 but < 3 SD below the mean OR performance on a task is > 1 but < 2 SD below the mean AND functional impact on instrumental activities. Examples include, but are not limited to: special education services at school (IEP, 504 plan, NOT Self-contained), unable to reach educational/occupational goals secondary to memory impairment, assistance needed completing tasks at home, scheduling/ attending appointments, secondary to memory impairment

© 2024 Goggin KP et al. *JAMA Network Open*.

Domain	Grading Source	Grading Rubric
		3: Performance on a task is > 3 SD below the mean OR performance on a task is > 1 but < 3 SD below the mean AND functional impact on self-care activities. Examples include, but are not limited to: unable to live independently due to memory impairment, unable to work due to memory impairment, self-contained classroom<br>4: Not applicable<br>5: Not applicable
Executive function deficit	Performance on neuropsychological testing of executive functions, including measures of cognitive flexibility/shifting, verbal fluency/initiation, working memory, and self-monitoring.	1: Performance on a task is > 1 but < 2 SD below the mean AND no functional impairment<br>2: Performance on a task is > 2 but < 3 SD below the mean OR performance on a task is > 1 but < 2 SD below the mean AND functional impact on instrumental activities. Examples include, but are not limited to: special education services at school (IEP, 504 plan, NOT Self-contained), unable to reach educational/occupational goals secondary to cognitive impairment, assistance needed completing tasks at home, scheduling/ attending appointments<br>3: Performance on a task is > 3 SD below the mean OR performance on a task is > 1 but < 3 SD below the mean AND functional impact in self-care activities. Examples include, but are not limited to: unable to live independently, unable to work, self-contained classroom<br>4: Not applicable<br>5: Not applicable
Visuospatial deficit	Performance on neuropsychological testing of fine-motor dexterity and visual-motor integration. Impairment not explained by visual deficits or slow processing speed.	1: Performance on a motor skills task is > 1 but < 2 SD below the mean AND no functional impairment<br>2: Performance on a motor skills task is > 2 but < 3 SD below the mean OR performance on a motor skills task is > 1 but < 2 SD below the mean AND functional impact in instrumental activities. Examples include, but are not limited to: motor weaknesses impact performance across other cognitive domains, unable to keep up with handwriting demands, unable to prepare meals secondary to motor impairment<br>3: Performance on a motor skills task is > 3 SD below the mean OR performance on a motor skills task is > 1 but < 3 SD below the mean AND functional impact in self-care activities. Examples include, but are not limited to: unable to dress self, unable to feed self, unable to bathe self<br>4: Not applicable<br>5: Not applicable

Neurocognitive assessments used in participants: Ataxia/fine motor dexterity (Digit-Symbol Coding, Grooved Pegboard, Trail Making Test Parts A and B, Symbol Search, Visual Memory); Cognitive disturbance – executive dysfunction (Trail Making Test Part B, Controlled Oral Word Association Test, Digit Span Backwards, Continuous Performance Test-II perseverations or commissions); Cognitive disturbance – processing speed (Symbol Search, Controlled Oral Word Association Test); Concentration impairment (Continuous Performance Test-II omissions or variability, Trail Making Test Part A, Symbol Search, Digit Span Forward); Memory impairment (Digit Span Forward, California Verbal Learning Test-II learning slope or long-delay free recall, Visual Selective Reminding)

© 2024 Goggin KP et al. *JAMA Network Open*.

eTable 5. Incidence of Chronic Health Conditions by History of Severe Sepsis During Therapy for Leukemia: Alternative Analysis Approach

	No Sepsis (N=598)		Sepsis (N=46)		
**Chronic Condition (Outcome)**	**n**	**(%)**	**n**	**(%)**	**p-value***
**Cardiopulmonary**					
Cardiomyopathy, right ventricular systolic dysfunction, right heart failure, myocardial infarction	14	(2.4%)	0	(0%)	0.817
Restrictive pulmonary deficit, pulmonary diffusion deficit	4	(0.6%)	0	(0%)	0.817
Severe restrictive or obstructive pulmonary deficit, pulmonary diffusion deficit (≥ grade 3)	3	(0.5%)	0	(0%)	0.852
**Renal**					
Severe chronic kidney disease (≥ grade 3)	2	(0.3%)	0	(0%)	0.852
**Neurological**					
Cerebrovascular accident	6	(1%)	0	(0%)	0.852
Cerebrovascular accident, neuromuscular disorder	6	(1%)	0	(0%)	0.852
Severe cerebrovascular accident, neuromuscular disorder, seizures (≥ grade 3)	15	(2.5%)	0	(0%)	0.817
**All primary analysis events** (cardiac, pulmonary, neurologic) combined	22	(3.7%)	0	(0%)	0.749
**Sarcopenia** (low muscle mass)	171	(28.5%)	13	(30.5%)	0.889

Models adjusted for propensity score weights including radiation exposure, cumulative doses of vincristine, intrathecal methotrexate, intravenous/oral methotrexate, high dose methotrexate, alkylating agents, anthracyclines, asparaginase, corticosteroids and epipodophyllotoxins, age at diagnosis, and year of diagnosis. *p-values adjusted for false discovery rate based on 21 comparisons

© 2024 Goggin KP et al. *JAMA Network Open*.

eTable 6. Neurocognitive Impairment by History of Severe Sepsis During Therapy for Leukemia: Alternative Analysis Approach

	No sepsis (N = 598)		Sepsis (N = 46)							
**Neurocognitive condition**	**n**	**(%)**	**n**	**(%)**	**uHR**	**95% CI**	***P****	**aHR**	**95% CI**	***P****
**Moderate to severe impairment (grade 2 or 3)**										
Attention impairment	143	(23.9%)	18	(42.1%)	2.39	(1.47-3.92)	0.0005	2.87	(2.37-3.47)	<0.0001
Processing speed deficit	41	(6.9%)	3	(5.7%)	1.33	(0.40-4.37)	0.641	1.07	(0.72-1.59)	0.726
Memory impairment	158	(26.4%)	19	(41.3%)	2.22	(1.35-3.64)	0.002	2.72	(2.23-3.34)	0.0003
Executive function impairment	187	(31.3%)	22	(47.8%)	1.79	(1.08-2.98)	0.025	2.44	(2.06-2.90)	<0.0001
Visuospatial impairment	68	(11.4%)	8	(17.4%)	1.96	(0.90-4.24)	0.089	1.41	(1.06-2.48)	0.019
Any severe cognitive impairment	310	(51.8%)	29	(63.0%)	1.77	(1.20-2.61)	0.004	1.82	(1.58-2.09)	<0.0001
**Any impairment (grade 1-3)**										
Attention impairment	257	(42.9%)	21	(48.5%)	1.80	(1.16-2.79)	0.009	1.71	(1.48-2.00)	<0.0001
Processing speed deficit	147	(24.6%)	15	(33.8%)	1.60	(0.85-2.99)	0.142	2.33	(1.93-2.81)	<0.0001
Memory impairment	333	(55.7%)	29	(63.0%)	1.78	(1.14-2.78)	0.011	1.58	(1.38-1.81)	<0.0001
Executive function impairment	370	(61.8%)	30	(65.2%)	1.74	(1.19-2.55)	0.005	1.49	(1.30-1.70)	<0.0001
Visuospatial impairment	108	(18.1%)	8	(17.4%)	1.56	(0.82-2.95)	0.180	1.01	(0.75-1.31)	0.950
Any cognitive impairment	499	(83.4%)	39	(84.7%)	1.57	(1.08-2.30)	0.018	1.17	(1.04-1.32)	0.007

uHR, Unadjusted Hazard Ratio; aHR, adjusted hazard ratio; 95% CI, 95% confidence interval. Models are adjusted for propensity score weights, including radiation exposure, cumulative doses of vincristine, intrathecal methotrexate, intravenous/oral methotrexate, high-dose methotrexate, alkylating agents, anthracyclines, asparaginase, corticosteroids and epipodophyllotoxins, age at diagnosis, and year of diagnosis. **P* values adjusted for false discovery rate based on 21 comparisons

© 2024 Goggin KP et al. *JAMA Network Open*.

eReferences

1. Ehrhardt MJ, Howell CR, Hale K, et al. Subsequent Breast Cancer in Female Childhood Cancer Survivors in the St Jude Lifetime Cohort Study (SJLIFE). *J Clin Oncol.* 2019;37(19):1647-1656.
2. Howell CR, Bjornard KL, Ness KK, et al. Cohort Profile: The St. Jude Lifetime Cohort Study (SJLIFE) for paediatric cancer survivors. *Int J Epidemiol.* 2021;50(1):39-49.
3. Hudson MM, Ehrhardt MJ, Bhakta N, et al. Approach for Classification and Severity Grading of Long-term and Late-Onset Health Events among Childhood Cancer Survivors in the St. Jude Lifetime Cohort. *Cancer Epidemiol Biomarkers Prev.* 2017;26(5):666-674.
4. St. Jude Children's Research Hospital. Establishment of a Lifetime Cohort of Adults Surviving Childhood Cancer (SJLIFE). September 26, 2008; ClinicalTrials.gov Identifier: NCT00760656. Available at: https://clinicaltrials.gov/ct2/show/NCT00760656. Accessed 04/15/2020.
5. Goldstein B, Giroir B, Randolph A, International Consensus Conference on Pediatric S. International pediatric sepsis consensus conference: definitions for sepsis and organ dysfunction in pediatrics. *Pediatr Crit Care Med.* 2005;6(1):2-8.
6. Thomas NJ, Shaffer ML, Willson DF, Shih MC, Curley MA. Defining acute lung disease in children with the oxygenation saturation index. *Pediatr Crit Care Med.* 2010;11(1):12-17.

© 2024 Goggin KP et al. *JAMA Network Open*.
