# Supplementary material for: Severe Sepsis During Treatment for Childhood Leukemia and Sequelae Among Adult Survivors
Source: JAMA Netw Open. 2024 Mar 18;7(3):e242727. doi: 10.1001/jamanetworkopen.2024.2727 (PMC10949094; doi:10.1001/jamanetworkopen.2024.2727)
Supplement: Supplement 2. — Data Sharing Statement [file jamanetwopen-e242727-s002.pdf]

Data Sharing Statement

Goggin. Severe Sepsis During Treatment for Childhood Leukemia and Sequelae Among Adult Survivors. *JAMA Netw Open*. Published March 18, 2024.
doi:10.1001/jamanetworkopen.2024.2727

Data
Data available: Yes
Data types: Deidentified participant data
How to access data: Deidentified data will be made available by reasonable request to the principal investigator
When available: With publication

Supporting Documents
Document types: None

Additional Information
Who can access the data: Deidentified data will be made available by reasonable request to the principal investigator
Types of analyses: Any reasonable academic not-for-profit purpose.
Mechanisms of data availability: After approval of protocol
Any additional restrictions: N/A
